# Infectious Disease: In Disaster’s Wake: Tsunami Lung

**DOI:** 10.1289/ehp.113-a734

**Published:** 2005-11

**Authors:** Carol Potera

When the Asian tsumani struck on 26 December 2004, health authorities braced for an onslaught of waterborne illnesses including malaria and cholera, which often follow such disasters. But saltwater flooded the freshwater breeding grounds of the mosquitoes that spread malaria, and relief agencies quickly distributed bottled water, thwarting a cholera epidemic. Instead, a type of aspiration pneumonia named “tsunami lung” emerged and afflicted some survivors.

Tsunami lung occurs when people being swept by tsunami waves inhale salt-water contaminated with mud and bacteria. The resulting pneumonia-like infections normally are treated with antibiotics. However, the 2004 tsunami “wiped out the medical infrastructure, and antibiotics were not available to treat infections in the early stages,” says David Systrom, a pulmonologist at Massachusetts General Hospital in Boston. Consequently, victims’ lung infections festered, entered the bloodstream, and spread to the brain, producing abscesses and neurological problems such as paralysis.

Systrom and colleagues volunteered to work on a medical disaster team with Project HOPE (Health Opportunities for People Everywhere) aboard the hospital ship U.S. Naval Ship *Mercy* off the coast of Banda Aceh, Sumatra. When they arrived three weeks after the tsunami hit, “we saw infections not seen in the United States since before the development of antibiotics,” says Systrom. Among them were about 25 cases of tsunami lung. “No one expected the number of tsunami lung cases we saw,” says Systrom. “It was not on the radar screen.”

The diagnosis of tsunami lung requires a chest radiograph and computed tomography scan of the brain to confirm abscesses. This sophisticated equipment was available on the hospital ship. “Only the most severe cases with central nervous system involvement made it to the ship,” says Systrom. The team suspects that hundreds of milder cases went unreported.

In the 23 June 2005 issue of the *New England Journal of Medicine*, the team describes the case of a 17-year-old girl who aspirated water and mud while engulfed by a wave and carried about half a mile. She developed pneumonia two weeks later and was treated at a local clinic with unknown medicines. A week later, the right side of her face drooped, her right arm and leg became paralyzed, and she stopped talking.

A chest radiograph revealed air and pus outside the lining of the lung (a condition known as hydropneumothorax), and a brain scan showed four abscesses. After the doctors treated her with a combination of intravenous antibiotics (imipenem until the stock of that drug ran out, then vancomycin, cef-tazadime, and metronidazole), her speech and facial movement recovered first. When she moved her right leg and arm for the first time, she “burst into peals of laughter,” according to the report. She was transferred to an International Committee of the Red Cross–Crescent field hospital. “I suspect she’ll fully recover,” says Sydney Cash, a neurologist at Massachusetts General Hospital and member of the team, who has since received pictures of her walking.

A combination of microbes likely contributes to tsunami lung, but no lab facility was available to culture and identify those found in the Indonesian patients before the *Mercy* arrived. However, in a letter published in the 4 April 2005 issue of *The Medical Journal of Australia*, Anthony Allworth, director of infectious diseases at Royal Brisbane and Women’s Hospital, describes culturing *Burkholderia pseudomallei* from two tsunami lung patients in a land-based hospital and *Nocardia* species from a third.

*B. pseudomallei* lives in the Asian soil and water. Mark Pasternack, an infectious disease specialist at Massachusetts General Hospital who also served on the *Mercy*, says, “You do not have to directly aspirate *Burkholderia* to produce pneumonia. . . . After the tsunami, people had soft tissue injuries from being forced into objects, so they could have gotten *Burkholderia* from wounds or aspiration.”

Cash echoes this thought: “Natural disasters produce odd combinations of pathogens and unexpected ways for the body to be damaged that lead to unexpected clinical circumstances. [Medical disaster physicians need to] keep an open mind and expect the unexpected.”

Could an infection like tsunami lung emerge in victims of Hurricane Katrina? Probably not, speculates Pasternack. Although the water sweeping the Gulf Coast area may have been contaminated, “it was not forced down peoples’ lungs by high-speed waves,” he says. Therefore, aspiration pneumonia and its complications are unlikely to appear commonly during the Gulf Coast relief efforts.

## Figures and Tables

**Figure:**
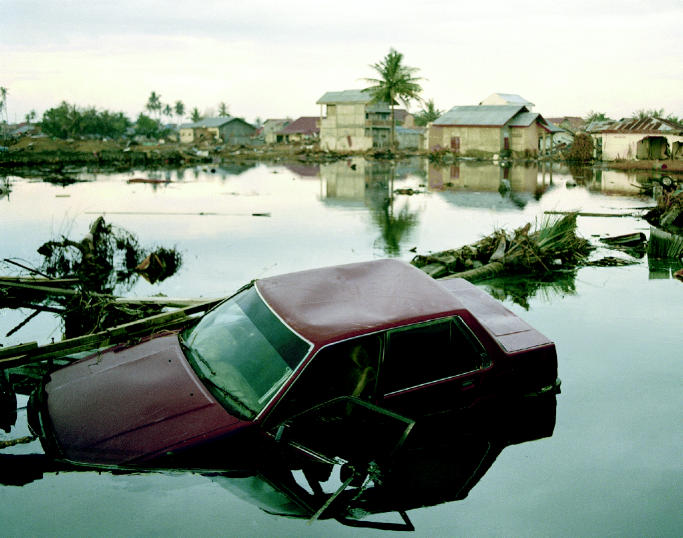
New concerns in devastation’s wake. Some survivors of the tsunami that struck South Asia on 26 December 2004 are experiencing a new peril—mud and bacteria they inhaled as they were swept along with the waves has led to a type of aspiration pneumonia called “tsunami lung.”

